# Transaction decision optimization of new electricity market based on virtual power plant participation and Stackelberg game

**DOI:** 10.1371/journal.pone.0284030

**Published:** 2023-04-20

**Authors:** Jinpeng Yang

**Affiliations:** School of Harbin Institute of Technology, Harbin Heilongjiang, Harbin, China; Xi’an Jiaotong University, CHINA

## Abstract

This study intends to optimize the trading decision-making strategy of the new electricity market with virtual power plants and improve the transmission efficiency of electricity resources. The current problems in China’s power market are analyzed from the perspective of virtual power plants, highlighting the necessity of reforming the power industry. The generation scheduling strategy is optimized in light of the market transaction decision based on the elemental power contract to enhance the effective transfer of power resources in virtual power plants. Ultimately, value distribution is balanced through virtual power plants to maximize the economic benefits. After 4 hours of simulation, the experimental data shows that 75 MWh of electricity is generated by the thermal power system, 100 MWh by the wind power system, and 200 MWh by the dispatchable load system. Comparatively, the new electricity market transaction model based on the virtual power plant has an actual generation capacity of 250MWh. In addition, the daily load power of the models of thermal power generation, wind power generation, and virtual power plant reported here are compared and analyzed. For a 4-hour simulation run, the thermal power generation system can provide 600 MW of load power, the wind power generation system can provide 730 MW of load power, and the virtual power plant-based power generation system can provide up to 1200 MW of load power. Therefore, the power generation performance of the model reported here is better than other power models. This study can potentially encourage a revised transaction model for the power industry market.

## 1. Introduction

### 1.1 Motivation and incitement

Energy is an essential basis for economic and social development. From the turn of the previous century, the world economy has expanded swiftly, while fossil fuel reserves have shrunk dramatically [1–3]. The worldwide lack of fossil fuels is a significant issue, and the tension between the reserve shortfall and the need for rapid economic development is becoming increasingly apparent. Meanwhile, pollution from using fossil fuels for energy worldwide is an issue for every nation. Using renewable energy sources has become an option for countries to combat the depletion of fossil fuels and the damage to the environment caused by combustion [4].

The power sector plays an integral role in the country’s long-term energy plan, and it is a vital aspect of the energy sector overall. The energy sector is widely viewed as the scope economy due to its inherent monopoly status [5]. The power industry has long implemented a monopoly model of vertical integration of power generation, transmission, distribution, and sales. However, the defects of the monopoly model of the power industry are gradually exposed with the continuous advancement of science and technology. Many countries have implemented structural adjustment and deregulation reforms in the energy industry to improve production efficiency. Market-oriented reforms have boosted competition in the power sector, reduced generation costs, improved service levels, and attracted the attention of the power industry. Many parts of the energy sector, however, have been presented with new obstacles and difficulties due to several technological and management issues that have surfaced during the reform process.

### 1.2 Literature review

This part compiles some of the recent research literature on electric power generation systems. Barik et al. (2022) [6] studied the recent trends and development process of hybrid microgrids. They analyzed the progress of energy resource planning and control research and discussed the prospects of energy management with the advantages of hybrid microgrids. The authors revealed that energy resource planning is crucial to renewable power’s international politics and economy. Ahmad et al. (2022) [7] investigated the determinants of renewable energy in Pakistan. Attempts were made to use renewable energy technology to address energy issues while keeping internal and external expenditures to a minimum. Their research can be a practical resource for fostering economic and energy integration in Pakistan. Tomin et al. (2022) [8] designed a flexible renewable energy based community microgrid system through an economic optimization approach. The study has practical applications for improving the quality of electricity supply. In summary, the efficiency of renewable energy supply can be promoted by optimizing the power system.

### 1.3 Contribution and paper organization

With the development of power interconnection and sharing technology, the traditional driving model for the power market needs to be optimized and reformed in the multi-subject energy power market transactions. However, the efficiency of the power system’s power resource production model is insufficient. For this reason, this study innovatively designs an open domestic power market equilibrium strategy algorithm. This study was inspired by a desire to refine the decision model for energy market transactions, as well as to encourage the development of models for virtual power plants and the improvement of photovoltaic power generation systems. The organization of the paper is summarized as follows. Section 1 introduces the background of the research on energy and virtual power plants. Section 2 optimizes the decision strategy of virtual power plants and power market trading. Section 3 analyzes the generation scheduling strategy and builds a market trading model. Section 4 analyzes the experimental results. Section 5 summarizes the research conclusions. This study has practical reference value for promoting the intelligent transformation of electric power generation systems.

## 2. Recent related work

### 2.1 Recent research on virtual power plants and Stackelberg game

This part provides a synopsis of some of the most important studies pertaining to virtual power plants. Li et al. (2018) [9] studied the decentralized collaborative scheduling method of multiple virtual power plants in the active distribution network. They proposed a two-level, decentralized stochastic scheduling model based on the simultaneous alternating direction multiplier method. A two-stage stochastic optimization method in the pre and re-scheduling stages was employed to enhance the stochastic uncertainty of distributed renewable energy output. The research results verified the effectiveness of the model. Yu et al. (2019) [10] explored virtual power plants’ uncertainty problems and countermeasures and introduced a distributed power generation system integrating multiple power sources. They categorized renewable electricity, market prices, and load demand as significant uncertainties and reviewed these factors comprehensively. The results indicated that the power system integrating multiple power sources could offer a reliable and friendly overall power supply.

In addition, many scholars have explored the blockchain and the Steinberg game. For example, Yang et al. (2021) [11] researched a blockchain-based residential distributed energy management system for a virtual power plant. They developed an energy management platform to implement a blockchain system and used a distributed optimization algorithm to manage the virtual power plant system. Panda et al. (2022) [12] devoted themselves to the conceptual overview of the transformation of microgrids into virtual power plants. They combined a virtual power plant with an integrated network of current smart power distribution. The results showed that integrating renewable energy is vital to intelligent power distribution. Li et al. (2021) [13] studied integrated energy systems with integrated demand response for renewable generation and developed a solution based on sequence operation theory. They found that the Stackelberg game-based method achieves a Stackelberg equilibrium between integrated energy operators and users by coordinating renewable energy generation. In addition, some scholars have worked on virtual power plants and sustainable energy management. Naval et al. (2021) [14] explored virtual power plant models and electricity market trading strategies. They combined multiple electricity purchasing and selling strategies. The results showed that the virtual power plant model could facilitate the power system operation and management capabilities. Barik et al. (2021) [15] researched distributed microgrid systems with sustainable energy sources by coordinating the voltage frequency of the controller. Their study can promote optimal resource allocation for hybrid microgrids with sustainable energy sources.

To sum up, a virtual power plant is defined as a kind of flexible cooperation between public utilities. Its establishment enables individual participants to provide customers with efficient services through the virtual sharing of their private property, improving individuals’ utilization efficiency and avoiding repeated construction.

### 2.2 Relevant research on transaction decision-making and optimization of the new electricity market

The spectrum domain confrontation mechanism can effectively optimize relevant trading decision-making and solve the equilibrium solution problem in the new power market. Qi et al. (2021) [16] studied the learning-based spectrum access Stackelberg game technology and designed a new spectrum domain confrontation mechanism. A parallel log-linear learning algorithm was proposed to search for the equilibrium solution efficiently. Each user intelligently decides their spectrum access strategy via this algorithm. The results showed that the algorithm is closer to the optimal solution than others. Zhang et al. (2022) [17] studied the dynamic pricing method of an integrated energy system based on the Stackelberg game. They established the Stackelberg game model of integrated energy system operators and integrated energy systems. The results verified that the integrated energy system brings new solutions for promoting energy coupling and improving energy efficiency.

Literature also exists on using deep reinforcement learning to solve decision-making issues. Liu et al. (2020) [18] studied data-driven decision-making strategies for electricity retailers through deep reinforcement learning. The findings demonstrated that the data-driven approach might solve the decision-making challenge and increase the retailers’ profits in the electricity market. Sun et al. (2021) [19] explored the combinatorial optimization strategy of retailer transactions in China’s electricity market. The scenario method was used to simulate random risk variables. Besides, an optimal decision-making model for power trading portfolios was established with the goal of profit maximization. The authors concluded that risk aversion factors in terms of purchase size and expected profits significantly influence the most fundamental trading patterns. Hu et al. (2021) [20] studied the bidding and quotation strategy of the Internet of Things microgrid and submitted a summary quotation to the market operator by coordinating each microgrid that joined. The authors optimized pricing and quotation strategies in electricity markets. Lu et al. (2022) [21] researched large electricity retailers’ medium- and long-term trading strategies in China’s electricity market. They optimized market trading strategies from the perspective of medium- and long-term contract electricity and benchmark price decisions. The research showed that many e-commerce companies and large consumers participate in electricity transactions, of which medium- and long-term transactions account for a large proportion in the current rapid advancement of the electricity spot market. Nasiri et al. (2022) [22] investigated the integrated multi-energy system of wind farms and considered various capacity issues, such as insufficient wind power production and overproduction. The study’s findings showed how effective wind-integrated multi-energy systems may be at mitigating hazards.

In sum, cooperative games are very different from non-cooperative games in that they place greater emphasis on communication within the alliance and the existence of legally binding agreements. Information exchange is the primary premise and basic capability for forming cooperation. It can encourage players with shared interests to ally with the same goal.

## 3. Market transaction model based on the Stackelberg game

### 3.1 Analysis of the generation dispatching strategy in China’s electricity market

**Fig 1:**
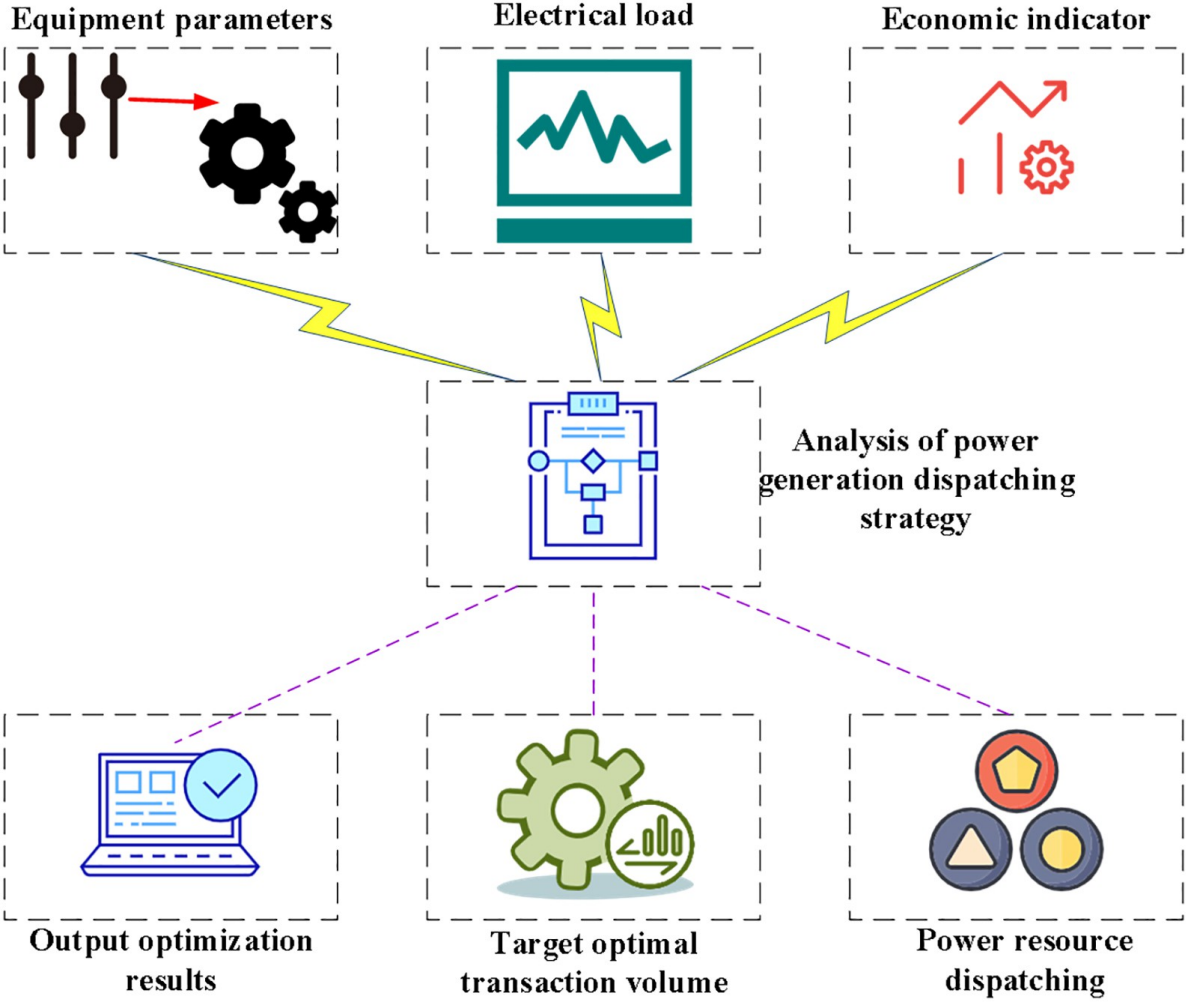
Structural analysis of the generation dispatch strategy in the electricity market.

Load forecast variation, unscheduled group power outages, the unpredictability of renewable energy, grid constraints, etc., all contribute to a deficiency of power resources in China’s direct electricity trading market. The lack of an effective market access mechanism between medium- and long-term electricity consumption and real-time energy balance [23] is essential in implementing long-term direct trade contracts. The monthly energy adjustment mechanism can meet the additional power demand by organizing the most economical generation dispatch strategy and reducing energy consumption per a predetermined dispatch sequence. Fig 1 depicts the structure of a generation dispatch strategy for the electricity market.

### 3.2 Electricity market transaction model under Stackelberg game

In the electricity market game model [24] with virtual power plants, the electricity demand is calculated through the utility maximization function *D*(*P*).


$$D(P)=\frac{C-\alpha P}{\theta }$$ (1)

In Eq (1), *D*(*P*) represents the system’s power demand; *C* stands for the user’s power consumption parameter; *α* signifies the price of power demand; *P* refers to the electricity sales price of the electricity sales company; *θ* denotes the given parameter.

Assuming that there are *N* electricity sales companies in the electricity trading market that are jointly responsible for the electricity sales business in this area, the total electricity demand $D_{i}^{x}\left(P^{\left(x\right)}\right)$ of electricity users is expressed as Eq (2).


$$D_{i}^{x}\left(P^{\left(x\right)}\right)=D_{i}^{x}-\alpha_{i}^{\left(x\right)}P_{i}^{\left(x\right)}+\sum_{n=1,n\neq i}^{N}\;v_{i,u}^{\left(x\right)}P_{i}^{\left(x\right)}$$ (2)

In Eq (2), *P*^(*x*)^ denotes the electricity sales price vector of all electricity sales companies; $D_{i}^{x}$ represents the parameter value due to time changes; $\alpha_{i}^{\left(x\right)}$ signifies the price elasticity of power resource demand; $P_{i}^{\left(x\right)}$ refers to the electricity sales price of companies in the region *x*; $v_{i,u}^{\left(x\right)}$ stands for the change ratio of power demand.

Eqs (3) and (4) describe the company’s profit function *Ri*(*P*) and profit coefficient *R*_1_(*P*_1_, *P*_*2*_).


$$R_{i}(P)=\sum_{x\in X}\;D_{i}^{\left(x\right)}\left(P^{\left(x\right)}\right)+M_{i}P_{i}$$ (3)

$$R_{1}\left(P_{1},P_{2}\right)=\sum_{x\in X}\;D_{i}^{\left(x\right)}\left(P_{1},P_{2}\right)P_{1}+M_{1}P$$ (4)

In Eqs (3) and (4), *x* stands for the regional collection of electricity sales company services; *M*_1_ denotes the number of users of large electricity sales companies; *P* refers to the fixed price of electricity sales; $\sum_{x\in X}\;D_{i}^{\left(x\right)}$ signifies the profit of the electricity market. The Nash equilibrium coefficient $P_{1}^{*}$ of the Stackelberg dynamic game is calculated by Eq (5) to count the electricity sales price of the new electricity market transaction.


$$P_{1}^{*}=\left\{\left(2E_{1}D^{\text{req}\;}M_{1}-2E_{1}\beta S_{1}\right)\sum_{x\in X}\;\left(N_{2}v_{1,2}^{\left(x\right)}G^{\left(y\right)}-\alpha_{1}^{\left(x\right)}\right)\right.$$ (5)

In Eq (5), *M*_1_ denotes the equilibrium coefficient of the first stage; *D*^req^ represents the dynamic game coefficient; *E*_1_ signifies the weighted constant coefficient of the discount provided by the power market; *β* stands for the realization factor of purchasing power; *S*_1_ represents all the electricity sold by the electricity sales company; *G*^(*y*^) refers to the unit cost of electricity purchased by the company. The effectiveness coefficient of the selling price strategy *G*^(*y*^) and the service risk index *S*_2_ (*P*_1_) are expressed as:

$$G^{\left(y\right)}=\frac{\left(2E_{2}\sum_{y\in Y}\;\alpha_{2}^{\left(y\right)}+M_{2}\right)N_{1}\sum_{y\in Y}\;v_{2,1}^{\left(y\right)}}{\left(2E_{2}\sum_{y\in Y}\;\alpha_{2}^{\left(y\right)}+2M_{2}\right)\sum_{y\in Y}\;v_{2}^{\left(y\right)}}$$ (6)

$$S_{2}\left(P_{1}\right)=F^{\left(y\right)}+G^{\left(y\prime \right)}P_{1}$$ (7)

where *M*_2_ indicates the number of users of small and medium-sized electricity sales companies; *N*_1_ refers to the power system load; *P*_1_ signifies the electricity demand forecast of the electricity market; *F*^(*y*^) signifies the contract decomposition power of the electricity market. The noise function $D_{2}^{\left(y\right)}\left(P_{1},P_{2}\right)$ and discount coefficient of the power system $P_{2}^{*}$ can be written as Eqs (8) and (9).


$$D_{2}^{\left(y\right)}\left(P_{1},P_{2}\right)=D_{2}^{\left(y\right)}-\alpha_{2}^{\left(y\right)}P_{2}+N_{1}v_{2,1}^{\left(y\right)}P_{1}$$ (8)

$$P_{2}^{*}=S_{2}\left(P_{1}^{*}\right)=F+GP_{1}^{*}$$ (9)

In Eqs (8) and (9), $D_{2}^{\left(y\right)}$ refers to the experience replay coefficient; $\alpha_{2}^{\left(y\right)}$ signifies the target sample set; $N_{1}v_{2,1}^{\left(y\right)}$ represents the marginal electricity price; $GP_{1}^{*}$ denotes the quotation of the generator. Eq (10) represents the final electricity sales price *P*_sn_(*r*) of the electricity sales company.


$$P_{sn}(r)=P_{n}(r)+P_{0}$$ (10)

In Eq (10), *r* denotes the electric energy utility grade index, *P*_0_ refers to the basic electricity price, and *P*_n_(*r*) represents the additional service price of electricity sales. The power supply cost *C*_n_(*r*) of electric energy generation is calculated via Eq (11).


$$C_{n}(r)=\alpha_{cn}r^{2}+\beta_{cn}r$$ (11)

In Eq (11), *α*_*cn*_ and *β*_*cn*_ represent the cost coefficient of power supply, and *r* stands for the utility level of electric energy. The length *d* and grade lower limit *r*_*x*_ of different intervals of power quality are calculated as follows:

$$d=\frac{1-r_{0}}{X}$$ (12)

$$r_{x}=r_{0}+xd$$ (13)

In Eqs (12) and (13), *r*_0_ represents the basic quality level of electric power, and *x* refers to the lower limit value of the level.

The individual user’s preference influence coefficient *g*_*mn*_ for different power companies and the average price *P*_*avn*_ of power quality are expressed as Eqs (14) and (15).


gmn=1,Pavn≤1+δmPRm,αmn=00,Pavn>1+δmPRm,αmn=0/1 (14)

$$P_{avn}=\frac{1}{X}\sum_{x=1}^{X}\;P_{sn}\left(r_{x}\right)$$ (15)

In Eqs (14) and (15), *α*_*mn*_ signifies the preference coefficient of power users for electricity sales companies, *P*_*Rm*_ denotes the user’s internal reference price, and *P*_*sn*_(*r*_*n*_) represents the power price coefficient for consumers’ reference.

The goal of the revenue model is to maximize the electricity transaction price *G*_*cn*_ acceptable to the market, as expressed by Eq (16).


$$maxG_{cn}\sum_{m=1}^{M}\;g_{mn}^{\prime }P_{sn}\left(r_{x}\right)-C_{n}\left(r_{x}\right)Q_{i}$$ (16)

In Eq (16), $g_{mn}^{\prime }$ denotes the revenue of the electricity sales company, and *Q*_*i*_ stands for the electricity purchase amount of the electricity user.

Eq (17) expresses the constraints on the user’s selection of the quality level of electric energy.


$$U_{m}\left(r_{x}\right)-P_{sn}\left(r_{x}\right)\geq U_{0}-P_{0},r_{x}\in \left\{r_{1},r_{2},\ldots ,r_{X}\right\}$$ (17)

**Fig 2:**
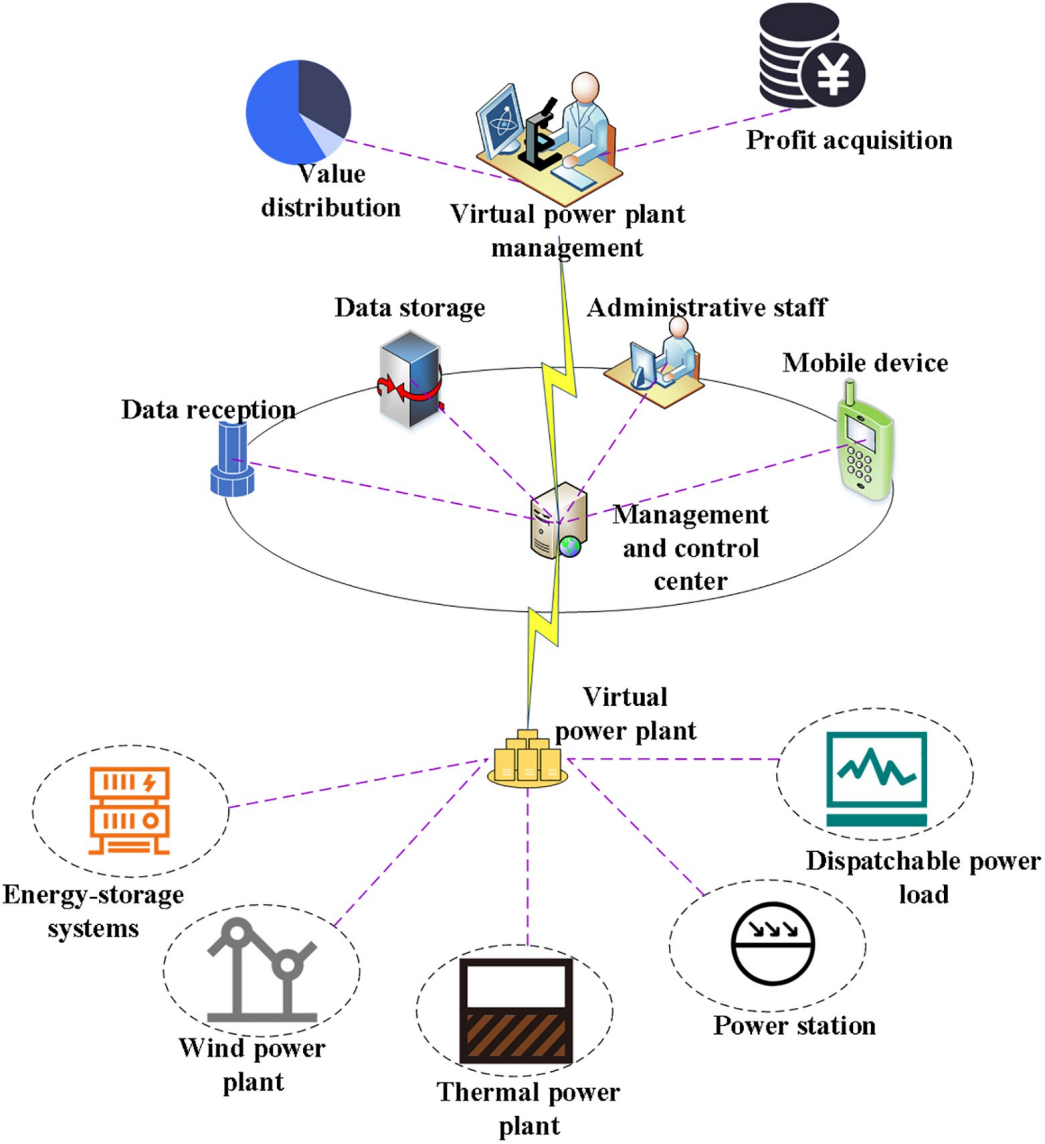
Structure of the electricity market transaction model under Stackelberg game.

In Eq (16), *U*_0_ refers to the basic power utility coefficient, *P*_0_ represents the corresponding electricity price, and *r*_*x*_ stands for the corresponding power quality index. Fig 2 illustrates the structure of the power market transaction model under the Stackelberg game.

### 3.3 Equilibrium value distribution strategy of virtual power plant based on cooperative game

In addition, Eq (18) indicates the optimization model of power user revenue in the context of cooperative games to maximize total revenue [25].


$$maxG_{umt}=\sum_{n=1}^{N}\;g_{mn}^{\prime }U_{m}\left(r_{x}\right)-P_{sn}\left(r_{x}\right)Q_{i}$$ (18)

In Eq (18), *G*_*umt*_ represents the income of power users, *U*_*m*_(*r*_*x*_) denotes the user income index.

The restrictive conditions for the power purchase cost of power users are expressed in Eq (19).


$$G_{cn}\left[ar_{1}+(1-a)r_{2}\right]\geq min\left\{G_{cn}\left(r_{1}\right),G_{cn}\left(r_{2}\right)\right\}$$ (19)

In Eq (19), *G*_*cn*_(*r*_1_) and *G*_*cn*_(*r*_2_) represent the classification function of electric energy utility level.

In addition, the objective function of power profit maximization for the new power market transaction decision-making model with the virtual power plant (NPMTDM-VPP) established here is expressed as Eq (20).


$$max\sum_{t=1}^{T}\;\left(\lambda_{t}P_{t}^{vpp}+\lambda^{-}P_{t}^{vpp^{-}}+U\left(P_{t}^{dl}\right)-\lambda^{+}P_{t}^{+vpp}-C_{t}^{vpp}\right)$$ (20)

In Eq (20), *t* stands for the serial number of the time period; *λ*_*t*_ denotes the unified power storage price in the market at time t; *λ*^+^ and *λ*^−^ represent the electricity sales price and purchase price between the virtual power plant and the main grid; $P_{t}^{vpp}$ signifies the output of the virtual power plant; $U\left(P_{t}^{dl}\right)$ refers to the power consumption of dispatchable load; $C_{t}^{vpp}$ signifies the total cost of the virtual power plant. These elements are calculated expressed as:

$$C_{t}^{vpp}=C_{t}^{g}+C_{t}^{w}+C_{t}^{pv}+C_{t}^{s}$$ (21)

$$C_{t}^{g}=\sum_{i_{g}=1}^{G}\;a_{g}{\left(P_{i_{g},t}^{g}\right)}^{2}+b_{g}P_{i_{g},t}^{g}+c_{g},\forall t,\forall i_{g}\in G$$ (22)

$$C_{t}^{w}=\sum_{i_{w}=1}^{W}\;a_{w}{\left(P_{i_{w},t}^{w}\right)}^{2}+b_{w}P_{i_{w},t}^{w}+c_{w},\forall t,\forall i_{w}\in W$$ (23)

$$C_{t}^{pv}=\sum_{i_{pv}=1}^{PV}\;a_{pv}{\left(P_{i_{pv},t}^{pv}\right)}^{2}+b_{pv}P_{i_{pv}}^{pv}+t+c_{pv},\forall t,\forall i_{pv}\in PV$$ (24)

$$C_{t}^{s}=\sum_{i_{s}=1}^{S}\;a_{s}\left(P_{i_{s},t}^{cha}+P_{i_{s},t}^{dis}\right),\forall t,\forall i_{s}\in S$$ (25)

$$U\left(P_{t}^{dl}\right)=\sum_{i_{dl}}^{DL}\;U\left(P_{i_{dl}}t^{dl}\right),\forall t,\forall i_{dl}\in DL$$ (26)

**Fig 3:**
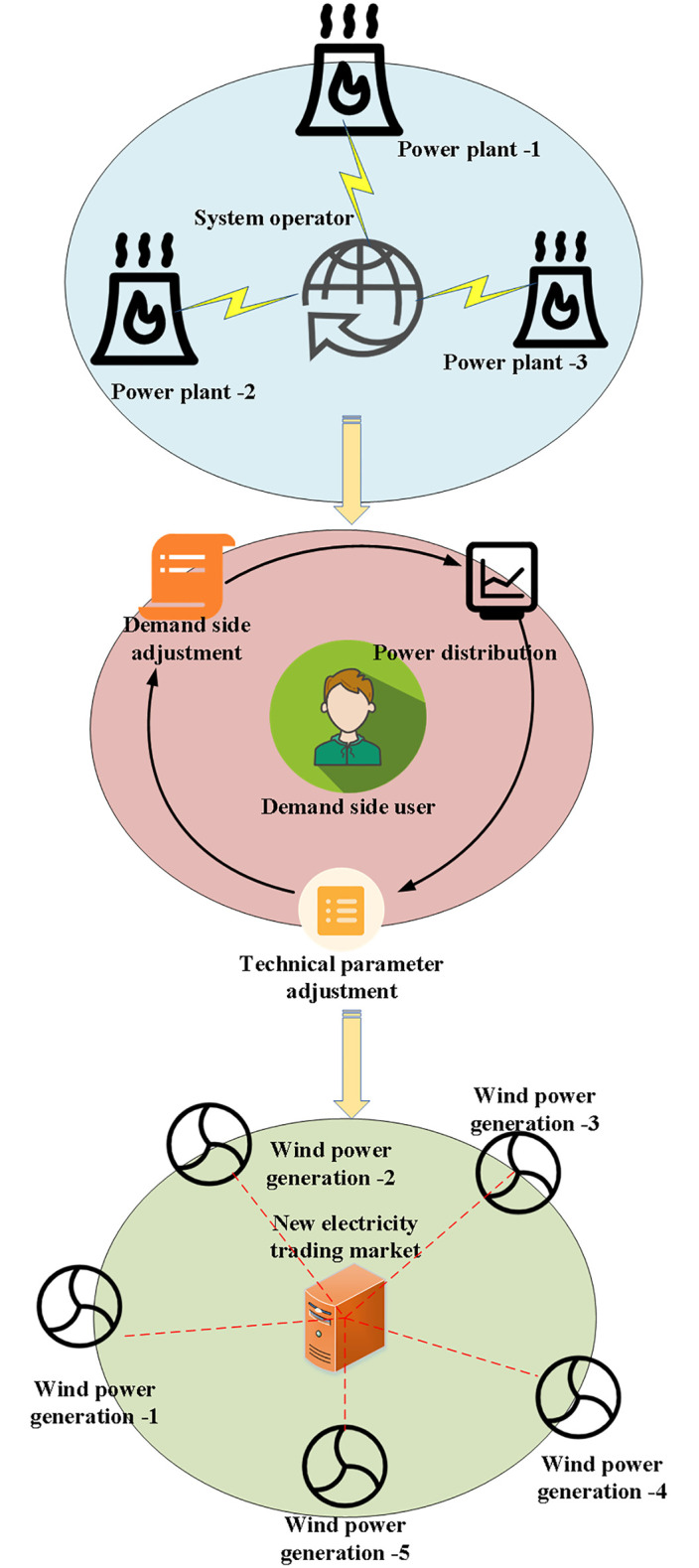
Equilibrium value distribution strategy of virtual power plant in cooperative game.

Where *i*_g_, *i*_*w*_, *i*_*pv*_, *i*_*s*_ and *i*_*dl*_ represent the numbers of thermal power plants, wind power plants, photovoltaic power plants, energy storage systems, and dispatchable power load systems; *G*, *W*, *PV*, *S* and *DL* denote the sets corresponding to each subject; $C_{t}^{g},C_{t}^{w},C_{t}^{pv}$ and $C_{t}^{s}$ refer to the cost of each output subject inside the virtual power plant; $P_{i_{g},t}^{g}$ represents the power generation cost of the thermal power plant; $P_{i_{w},t}^{w}$ and $P_{i_{pv}}^{pv}$ represent the power generation costs of wind power plants and photovoltaic power plants, respectively; *a*_*s*_ refers to the energy storage cost coefficient; $\sum_{i_{dl}}^{DL}\;U\left(P_{i_{dl}}t^{dl}\right)$ represents the *i*_*dl*_-th utility coefficient of the dispatchable load at the time *t*^*dl*^. Fig 3 illustrates the equilibrium value distribution strategy of the virtual power plant in the cooperative game.

### 3.4 Modeling and model analysis of virtual power plant

**Fig 4:**
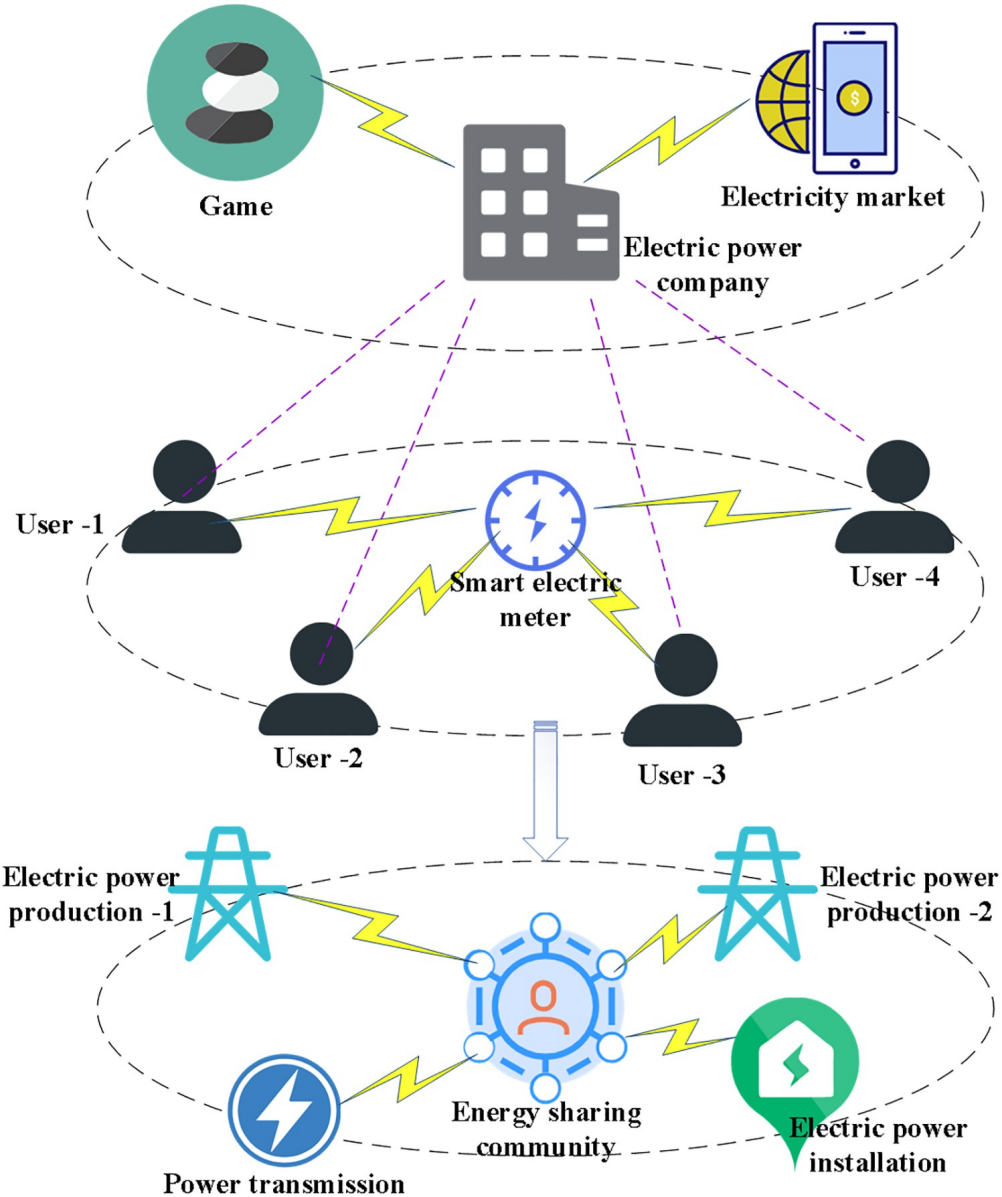
Modeling of virtual power plant.

Dispatchable load and storage system capacity are relatively small in virtual power plants. In particular, they are both consumers and producers at a specific moment. The energy storage system is mainly used to support the use of new energy in the virtual power plant, and the actual power generation capacity is limited. The energy storage system can obtain the maximum benefit through the time-sharing charging and discharging strategy. At the same time, this study collects actual historical data from PV plants and performs pre-processing operations for factors such as PV power, temperature, wind speed, and radiation dispersion levels. After completing the parameter tuning operation, the resultant data set is saved. All process data sets are available in the whole experimental session. The statistical analysis of the data includes filtering, pre-processing, and statistical correlation analysis. In addition, it is necessary to compare the power generation and cost of systems with different power loads, such as thermal power generation systems, wind power generation systems, photovoltaic power generation systems, load storage systems, dispatchable load systems, and virtual power plants. Fig 4 displays the structure of the established virtual power plant.

## 4. Results and discussion

### 4.1 Comparison of power performance of different power load systems

**Fig 5:**
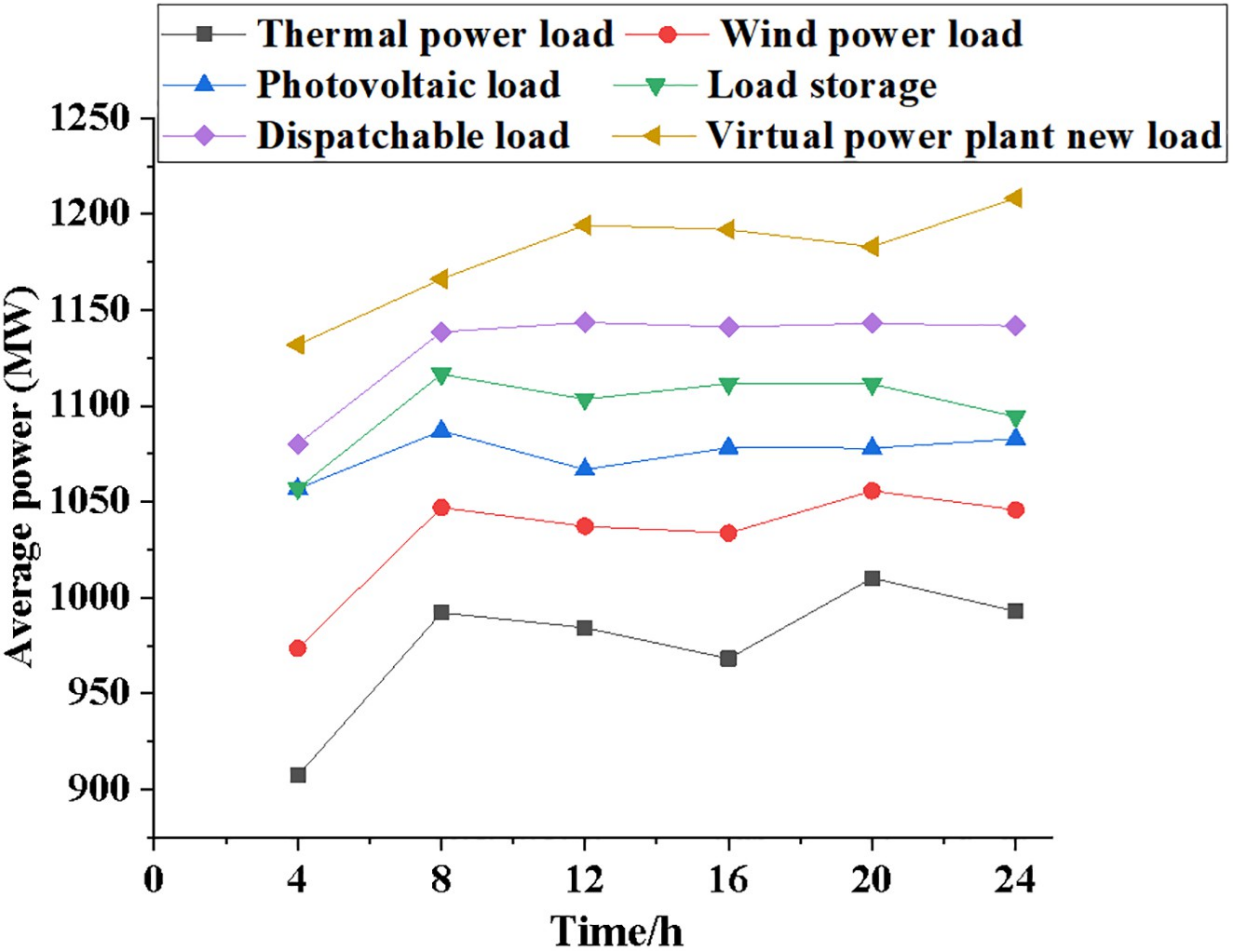
Comparison of average power changes of different electric load types.

**Fig 6:**
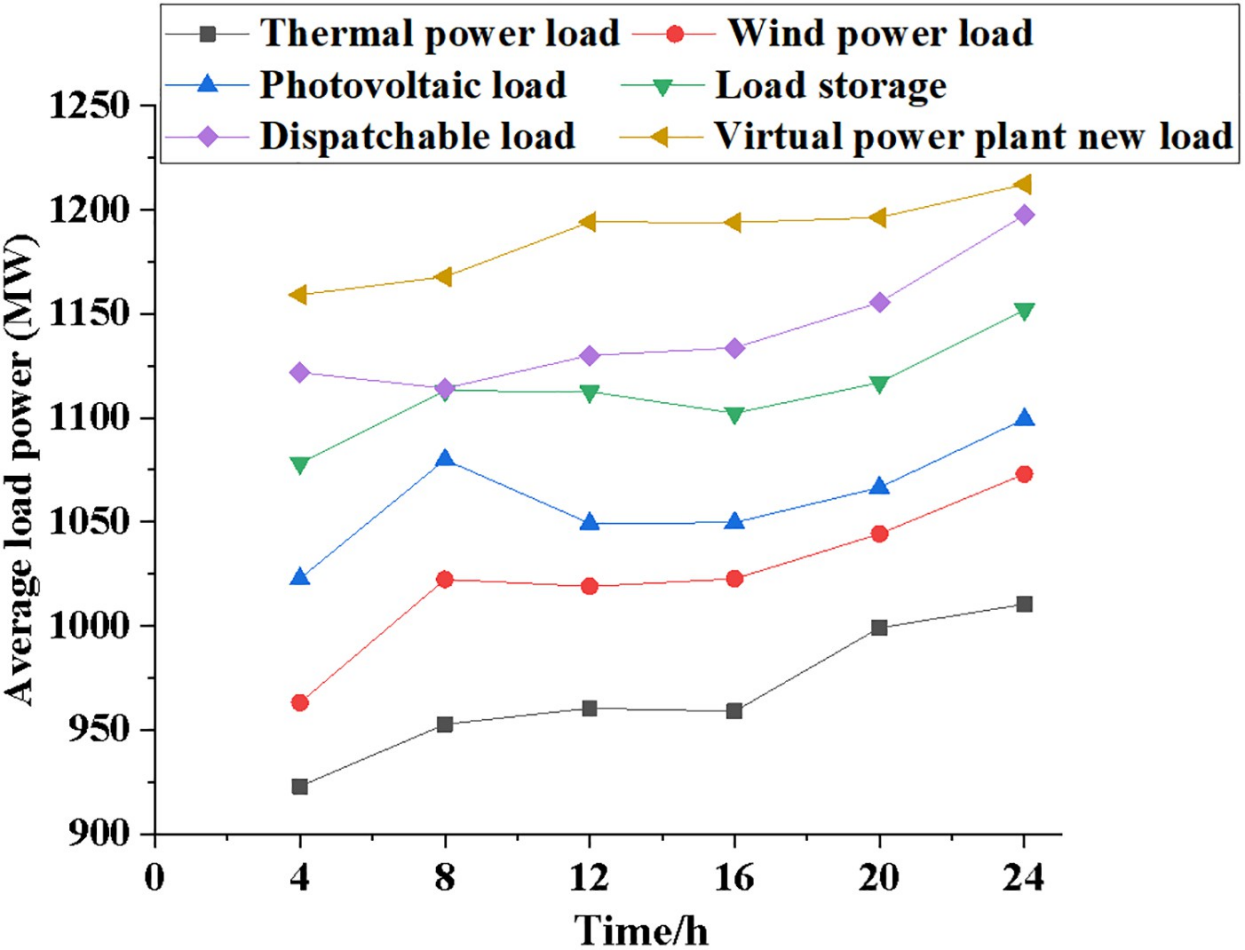
Change and comparison of average load power of different electric load types.

The performance of the NPMTDM-VPP model is compared with other electricity load systems. Fig 5 reveals the results of the average power variation of the six electric load systems as time increases. Figs 6~8 show information regarding the average load power change, the typical daily load power of different kinds of electrical loads, and the forecast data of system power generation for different types of electrical loads.

**Fig 7:**
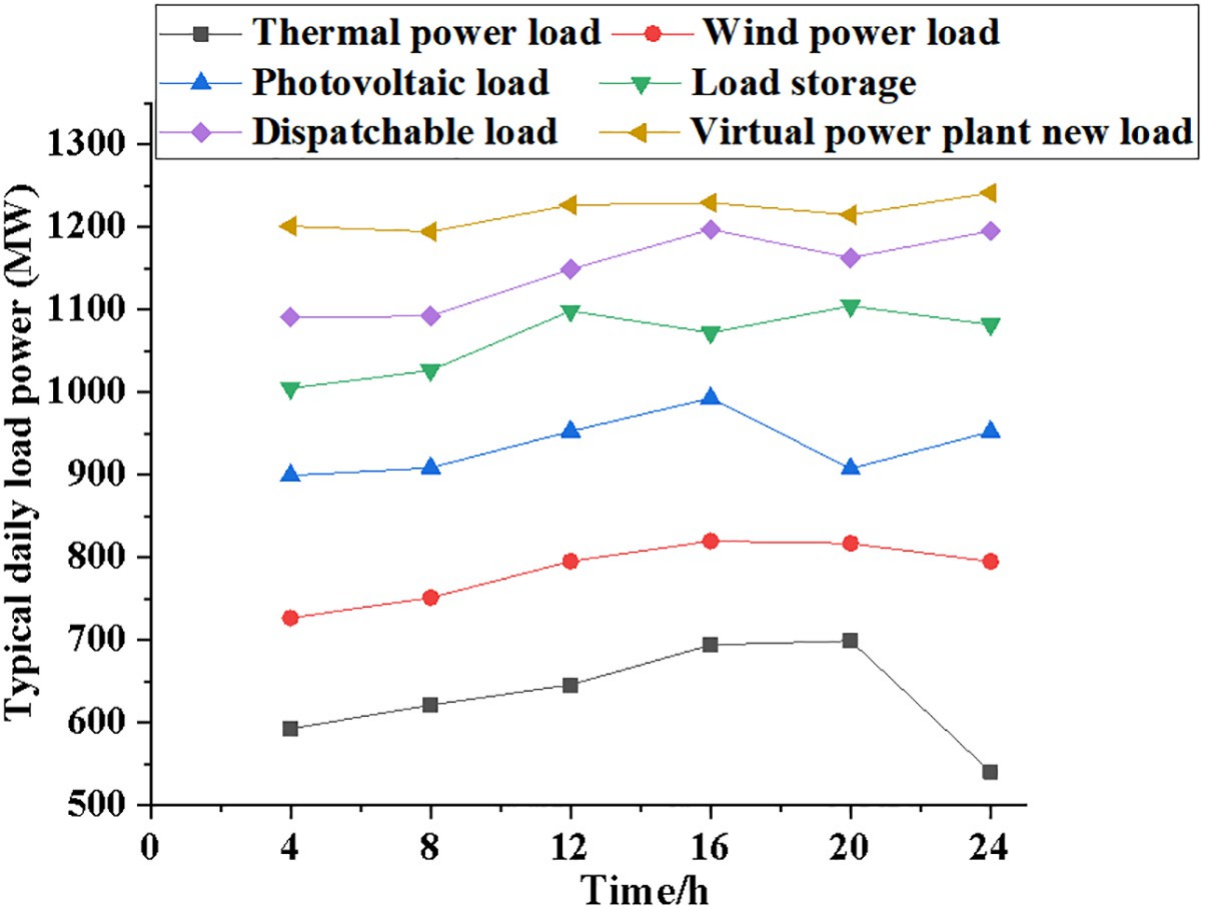
Typical daily load power comparison of different electric load types.

**Fig 8:**
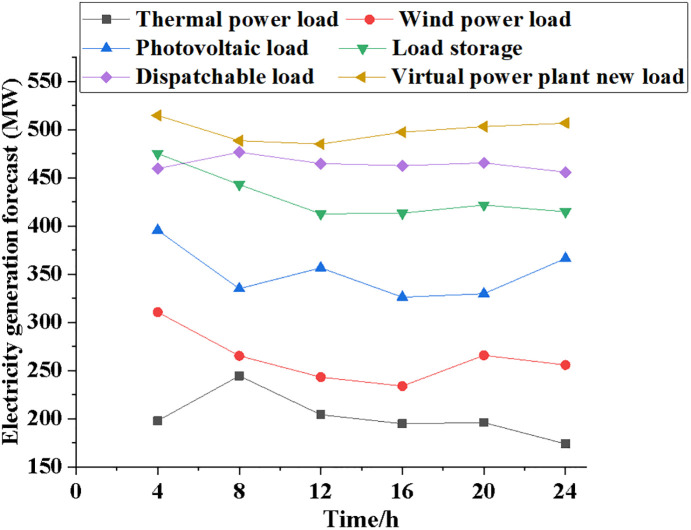
Variation trend of the forecast data of generated power for different power load types.

It can be seen from Fig 5 that the average power of different power load systems has certain changes and fluctuations with the passage of time. After 4 hours, the average power output of the thermal generation system is 900 MW, that of the dispatchable system is 1075 MW, and that of the NPMTDM-VPP model is 1125 MW. After 20 hours, the average power increase of the NPMTDM-VPP model is 1180 MW. After 24 hours, the average power increase of the NPMTDM-VPP model is 1215 MW. Therefore, the power market trading strategy can be optimized according to the power system’s load in different time periods.

According to Fig 6, the average load power of different electric load types varies dramatically with time growth. After 4 hours of simulation, the thermal power generation system averaged a load power of 925 MW; the wind power generation system has an average load power of 965 MW; there is an average load power of 1125 MW on the dispatchable power system; NPMTDM-VPP estimates an average load power of 1150 MW. In addition, the average load power of power systems with different load types will also show a upward trend over time. Throughout the course of a day, the average load power of a thermal power generation system is 1000 MW, of a wind power generation system is 1050 MW, and of a dispatchable power system or virtual power plant is above 1200 MW.

Fig 7 suggests that among several different power load systems, the typical daily load power of thermal power generation systems tends to rise first and then fall. In contrast, the daily load power of other types of power load systems basically shows an upward trend. The average daily load power of the NPMTDM-VPP model can reach 1200 MW, while the thermal power generation system has a daily load power of 600 MW and the wind power generation system has a daily load power of 730 MW when the time is 4 hours. After 20 hours, the typical daily load power of the thermal power generation system can reach 650 MW, and the average daily load power of the NPMTDM-VPP model is 1210 MW.

According to Fig 8, the power generation of different power load systems generally tends to decrease first and then rise. After 4 hours of operation, the virtual power plant is predicted to have 525MW of power, the thermal power system to have 200MW, the wind power system to have 320MW, the dispatchable power system to have 475MW of average load power, and the wind power system to have 320MW of power. In addition, after 24 hours, the power generation of the thermal power generation system is predicted to be 175 MW, while the power generation of the NPMTDM-VPP model can reach 520 MW. As a result, different electricity market trading decisions can be made at different times.

### 4.2 Comparison of power generation capacity and power generation cost of different power load systems

**Fig 9:**
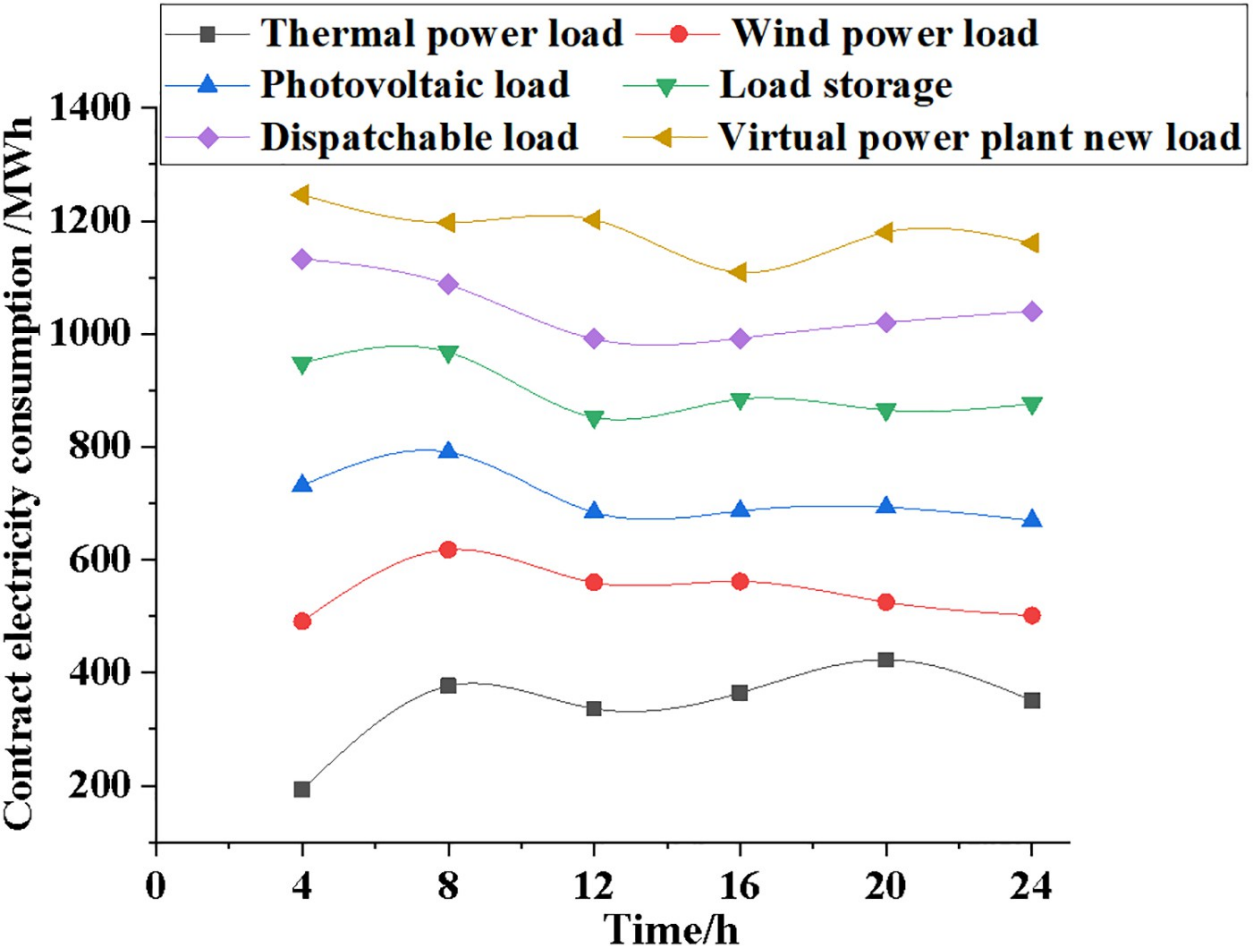
Changes and comparison of contract power data for different power load types.

Power contracts, actual generation data, marginal generation costs, and total generation costs are used to compare the generation and generation costs of systems with varying electric loads, sincluding those using thermal power, wind power, photovoltaic power, load storage, dispatchable load, and virtual power plants. Figs 9~12 illustrate the specific results.

**Fig 10:**
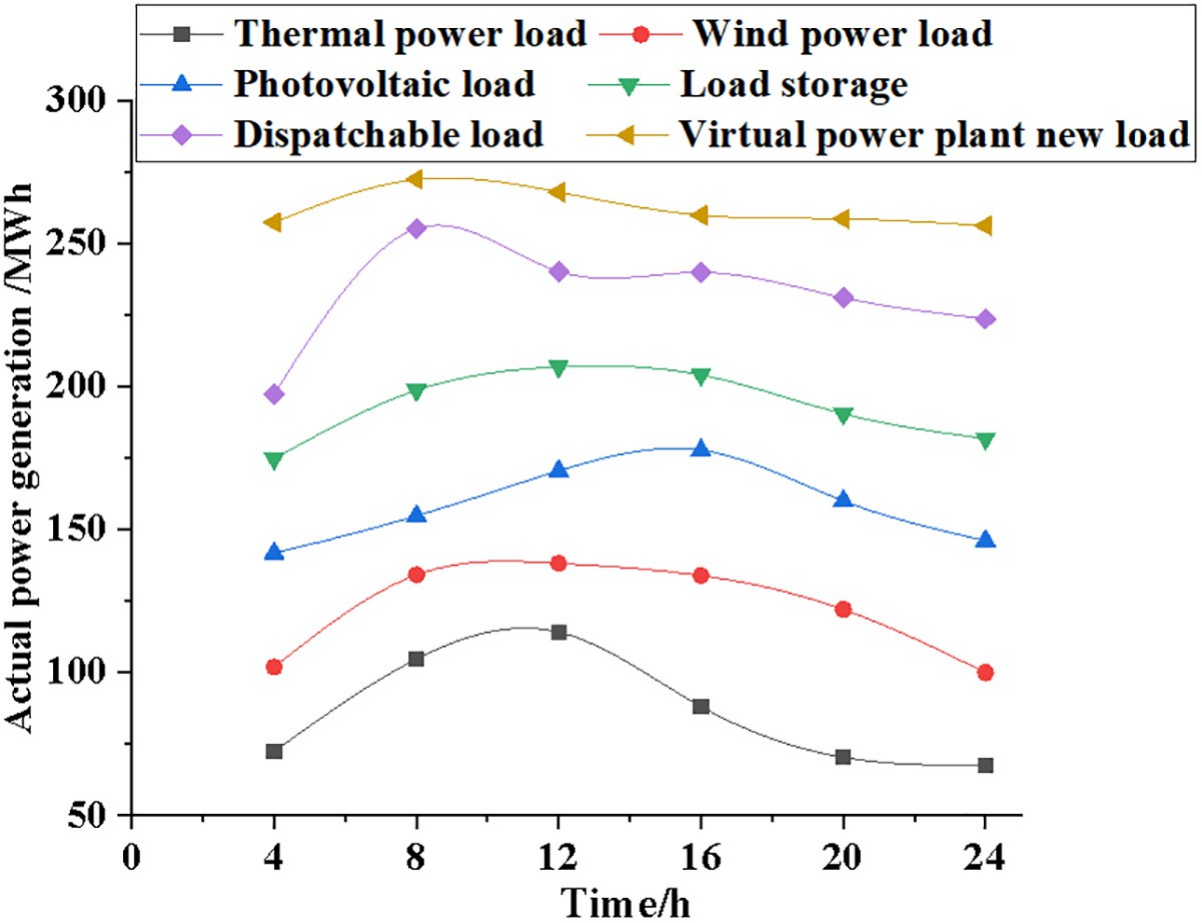
Change trend of actual power generation data of different power load types.

**Fig 11:**
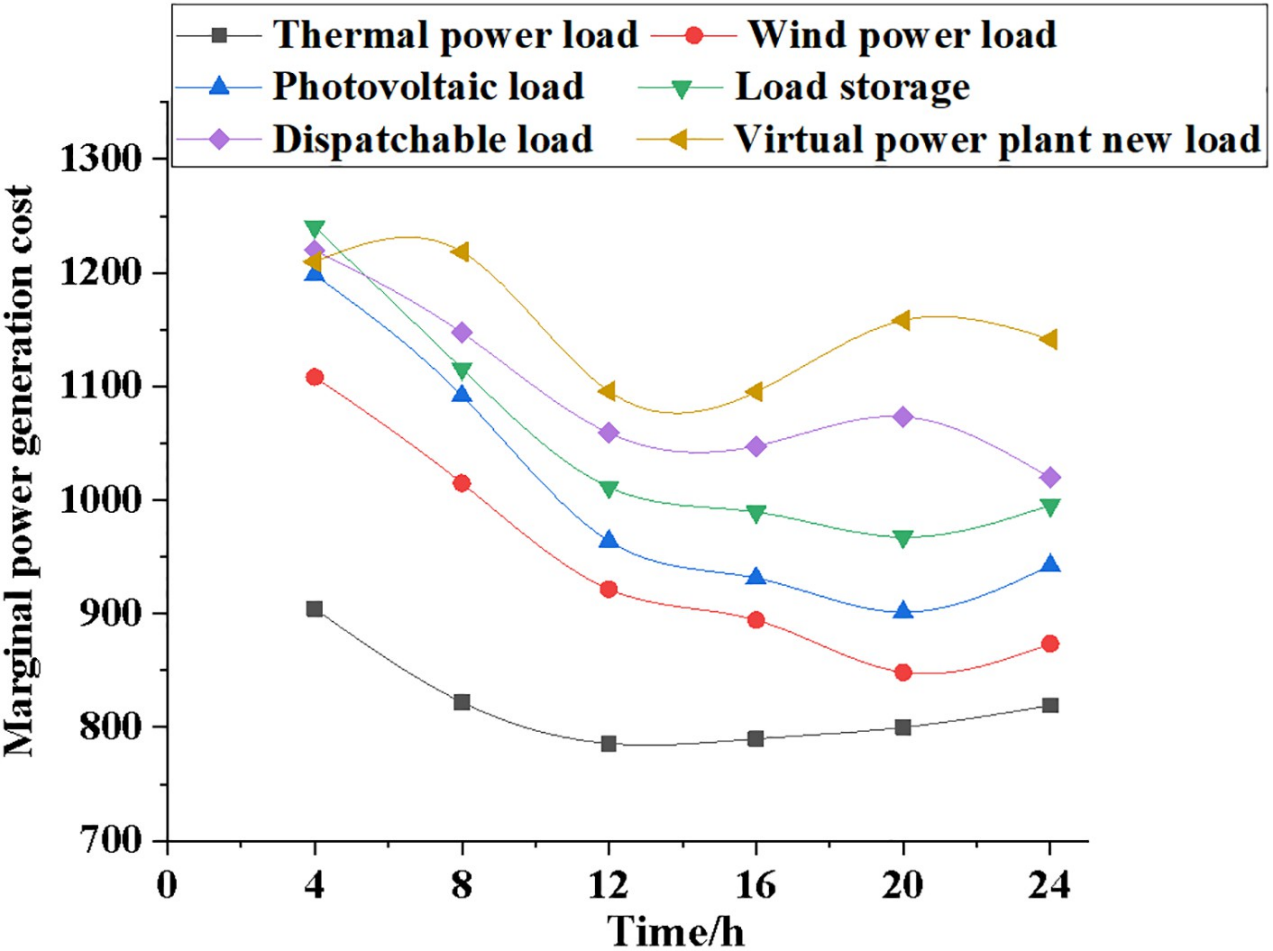
Comparison of marginal generation costs for different types of electrical loads.

**Fig 12:**
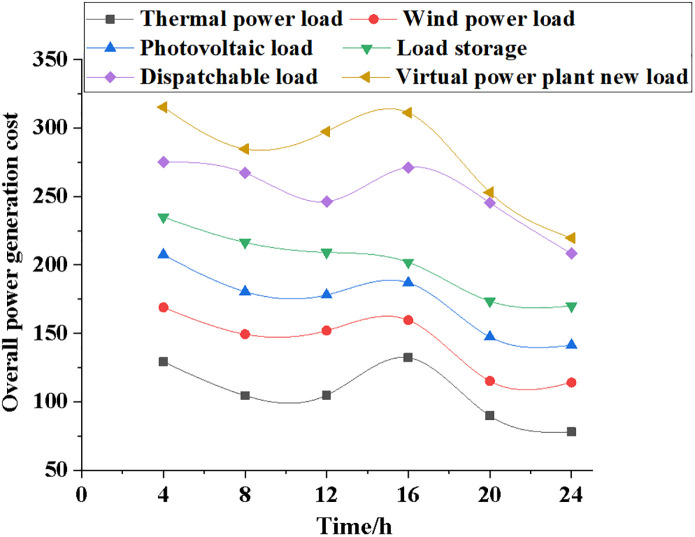
Comparison and analysis of total power generation costs for different power load types.

As can be observed in Fig 9, the contract power of the system will alter to some degree under various power load types, but will always remain within a set range. Contract power consumption for the thermal power plant is 200 MWh, for the wind power generation system it is 500 MWh, for the dispatchable storage power system it is 1120 MWh, and for the NPMTDM-VPP model it is 1290 MWh when the time is 4 hours. After 24 hours of simulation, the contract power consumption of the thermal power generation system can reach 400 MWh, the contract power consumption of the wind power generation system is 500 MWh, and the contract power consumption of the NPMTDM-VPP model may reach more than 1200 MWh. Therefore, the transaction decision of the electricity market can be reasonably optimized according to the data changes of the contracted electricity consumption.

Fig 10 indicates that under different power load types, the actual power generation data of the new power market will fluctuate significantly with the growth of time. After 4 hours of simulation, the NPMTDM-VPP model generates a total of 250 MWh, with 75 MWh coming from the thermal power generation system, 100 MWh from the wind power generation system, 200 MWh from the dispatchable load system, and 25 MWh from the renewable energy system. After 24 hours of simulation, the NPMTDM-VPP model generates 260 MWh, while the thermal power system generates 75 MWh, and the wind power system generates 100 MWh. As a result, the system’s power load can be met by time-of-day-specific resource allocation of the power actually generated.

It can be seen from Fig 11 that the marginal power generation cost of different power load types will first decrease and then rise with the model’s running time. After the model runs for 4 hours, the marginal power generation cost of the thermal power system is 900; the marginal power generation cost of the wind power system is 1100; the marginal power generation cost of the NPMTDM-VPP model is 1200. After the model runs for 24 hours, the marginal power generation cost of the thermal power generation system is 850; the marginal power generation cost of the wind power system is 900; the marginal power generation cost of the NPMTDM-VPP model is 1170. Therefore, the marginal generation cost of the model can be reduced according to the strategic transformation of different power systems.

Fig 12 suggests that in the total power generation cost of model operation, the power generation cost of the NPMTDM-VPP model is the highest, while the power generation cost of the thermal power generation system is the lowest. After the model runs for 4 hours, the power generation cost of the thermal power generation system is 125; the total power generation cost of the wind power system is 175; the total power generation cost of the NPMTDM-VPP model is 325. In addition, after the model runs for 24 hours, the power generation cost of the NPMTDM-VPP model drops to 250. Therefore, the adjusted unit generation plan can minimize the total generation cost of the system according to the different power load types of the model.

### 4.3 Comparison of power resource allocation performance of different power load systems

**Fig 13:**
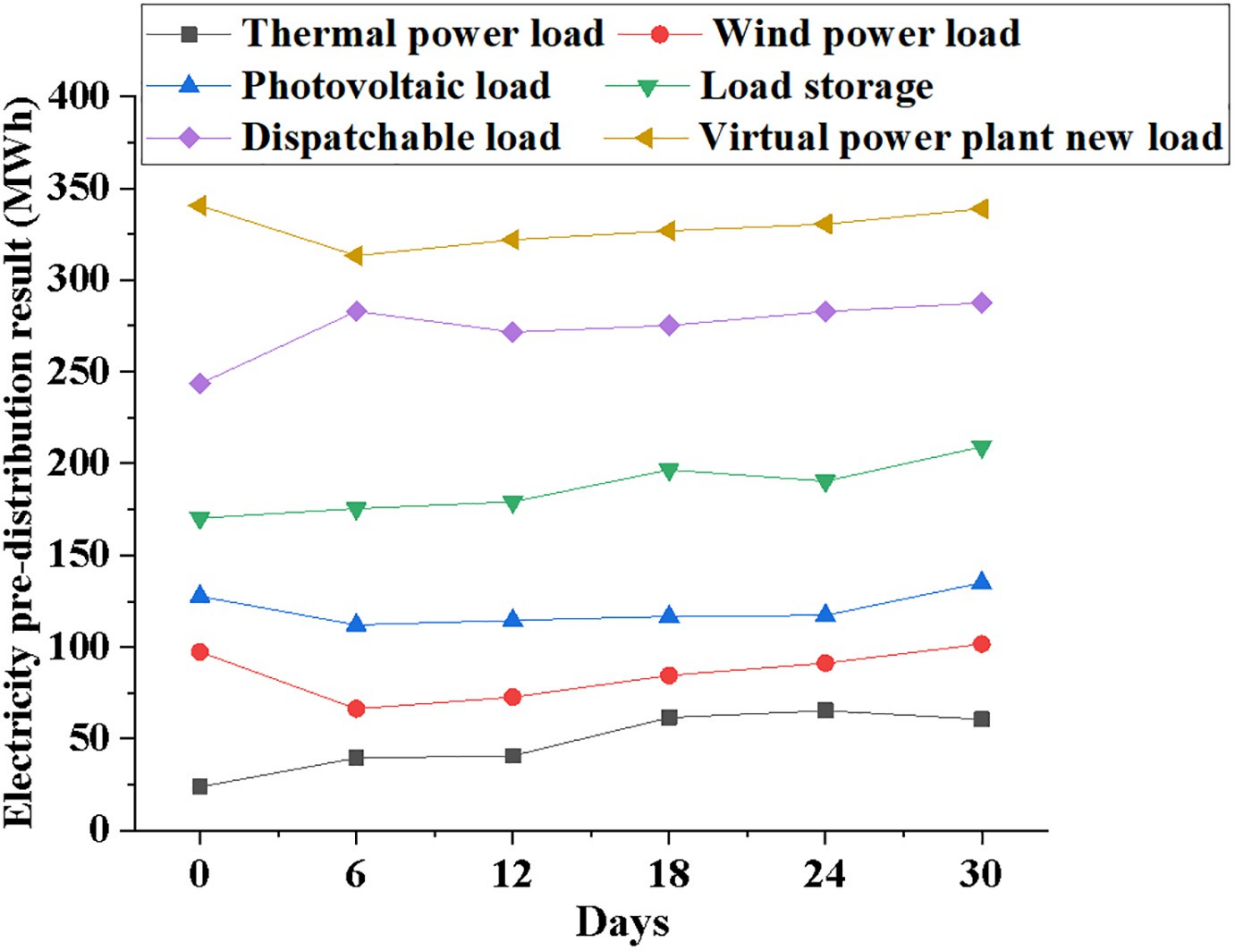
Variation curve of power pre-allocation results of different power load systems with time growth.

**Fig 14:**
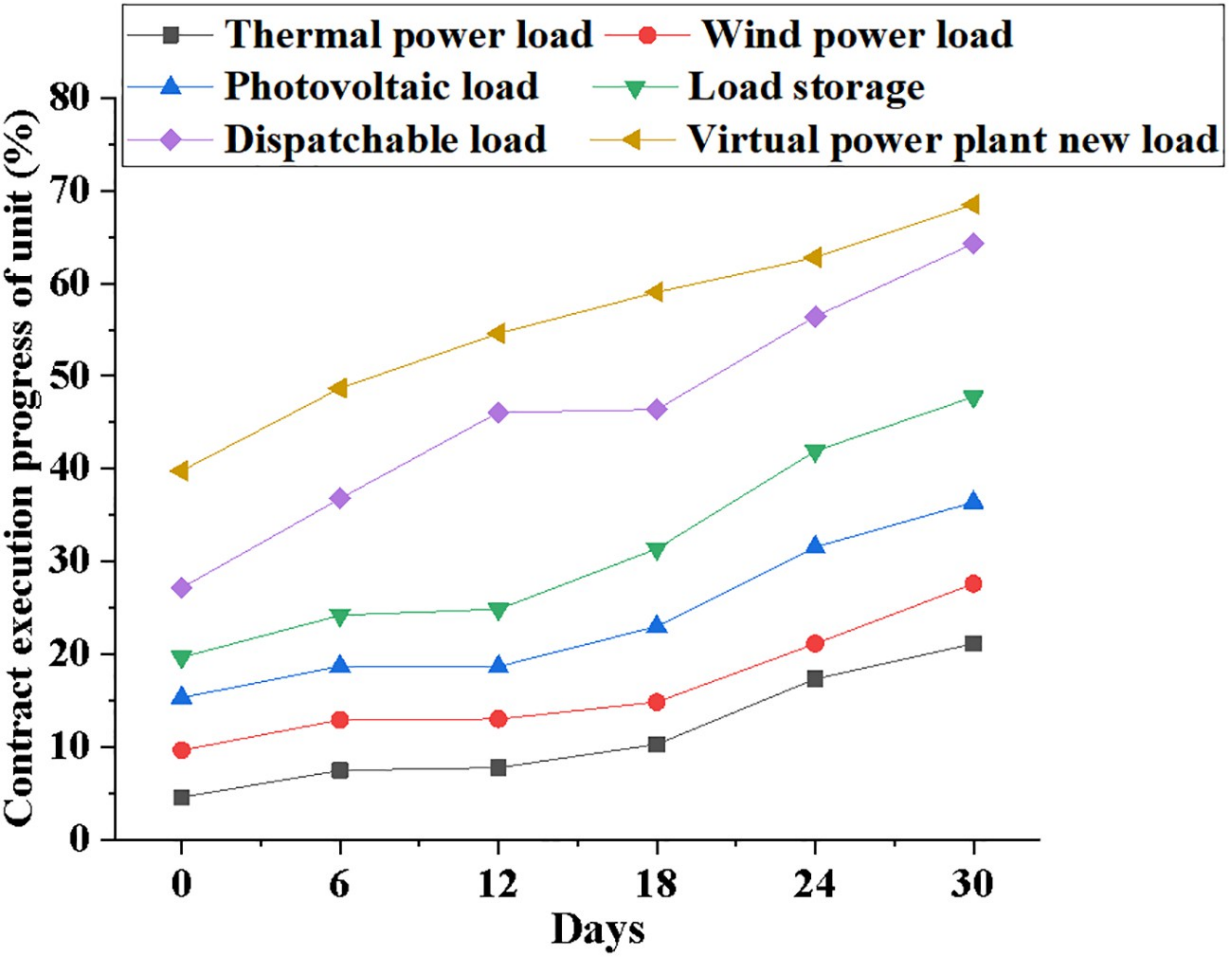
Curves of unit contract execution progress with time for systems with different power loads.

Figs 13 and 14 compare the power pre-allocation results and unit contract execution progress of different power load systems when the time parameter increases.

According to Fig 13, the resource pre-allocation results of systems with different power loads will produce inevitable fluctuations. When the model first started running, the pre-allocation consequence of the thermal power generation system is 25 MWh; the pre-allocation result of the wind power generation system is 100 MWh. Currently, the power pre-allocation result of the NPMTDM-VPP model is 350 MWh. After the model runs for 30 days, the pre-allocation result of the thermal power generation system is 75 MWh; the resource pre-allocation result of the NPMTDM-VPP model is 352 MWh. Therefore, the system’s power distribution outcomes can inform adjustments to various sorts of power resources.

It can be seen from Fig 14 that the execution progress of unit contracts of systems with different power loads will gradually increase. At the beginning of the model operation, the execution progress of the unit contract of the thermal power generation system is 5%; the execution progress of the unit contract of the wind power generation system is 10%; the execution progress of the unit contract of the NPMTDM-VPP model is 40%. After the model runs for 30 days, the unit contract execution progress of the NPMTDM-VPP model can reach 70%.

## 5. Discussion

This study analyzes different electric power generation scenarios. Compared with thermal, wind, and traditional photovoltaic power generation systems, the virtual power plant-based electric load system has higher power generation, lower power generation cost, and better actual power generation performance. Existing studies have shown that the system performance can be improved through the optimization and structural analysis of different power systems. Eslami et al. (2010) [26] examined the power system stabilizer rectified by the particle swarm algorithm. They optimized the objective function with constraints through the multi-objective particle swarm optimization method. The results showed that this scheme is a better optimization technique in terms of accuracy and convergence compared to particle swarm and genetic algorithms. Eslami et al. (2011) [27] examined the power system oscillation damping controller and optimized the power system’s weak contact network structure by considering dynamic stability factors. They concluded that the stability of the power system is the most critical aspect of the operation. Eslami et al. (2012) [28] developed a coordinated design strategy for the damped controller. Experiments proved that the controller scheme could significantly improve the dynamic stability of the power system through a novel heuristic global optimization algorithm.

## 6. Conclusion

The simultaneous pursuit of carbon neutrality and peak carbon dioxide emissions, made more urgent by the brisk expansion of the economy and society, calls for a sustained effort to create alternative energy sources. Therefore, a renewable, clean energy source has received extensive attention. This study optimizes the transaction decision-making strategy for the new electricity market against the background of virtual power plant participation. A transaction model of the electricity market under the Stackelberg game is proposed by analyzing the problems in China’s electricity market. The utility maximization function is utilized in the estimation of electrical consumption. This study compares the performance of the thermal power system, wind power system, photovoltaic power system, load storage system, dispatchable load system, and virtual power plant power system. The results showed that the power generation of different power load systems generally tends to decrease before increasing. When the model runs for 4 hours, the generation capacity of the thermal system is measured to be 200 MW; the generation capacity of the wind system is estimated to be 320 MW; the average load power of the virtual power plant system is predicted to be 475 MW. However, there are certain shortcomings in this study. The focus of research on virtual power plants and electricity market transactions here is the Chinese market. According to the scope of the study of virtual power plants and electricity market transactions, Future research will make adjustments to the inter-market game strategy in an integrated manner to optimize the trading environment of the global energy market.

## Supporting information

S1 Data (ZIP)
